# Novel Swine Influenza Virus Subtype H3N1, United States

**DOI:** 10.3201/eid1205.051060

**Published:** 2006-05

**Authors:** Porntippa Lekcharoensuk, Kelly M. Lager, Ramesh Vemulapalli, Mary Woodruff, Amy L. Vincent, Jürgen A. Richt

**Affiliations:** * US Department of Agriculture, Ames, Iowa, USA;; † Purdue University, West Lafayette, Indiana, USA

**Keywords:** Influenza A virus, swine influenza, novel H3N1 subtype, emerging disease of swine, research

## Abstract

Influenza A virus infects various animal species and transmits among different hosts, especially between humans and swine. Swine may serve as a mixing vessel to create new reassortants that could infect humans. Thus, monitoring and characterizing influenza viruses in swine are important in preventing interspecies transmission. We report the emergence and characterization of a novel H3N1 subtype of swine influenza virus (SIV) in the United States. Phylogenetic analysis showed that the H3N1 SIVs may have acquired the hemagglutinin gene from an H3N2 turkey isolate, the neuraminidase gene from a human H1N1 isolate, and the remaining genes from currently circulating SIVs. The H3N1 SIVs were antigenically related to the turkey virus. Lung lesions and nasal shedding occurred in swine infected with the H3N1 SIVs, suggesting the potential to transmit among swine and to humans. Further surveillance will help determine whether this novel subtype will continue to circulate in swine populations.

## Abstract 2

A new subtype H3N1 may have arisen from reassortment of a H3N2 turkey isolate, a human H1N1 isolate, and currently circulating swine viruses.

Influenza A viruses infect many animal species including birds, seals, whales, humans, horses, and swine. Migrating waterfowl are the primordial reservoir. They contain a gene pool of all subtypes of influenza A viruses (*1*), and phylogenetic analysis suggests that transmission of influenza A virus among various species can occur. Interspecies transmission between humans and swine has been documented (*1*). Both human and swine influenza viruses (SIVs) recognize sialyl α2,6-galactose oligosaccharide side chains as the receptor on the host cell surface (*2**,**3*). In addition, swine cells also contain sialyl α2,3-galactose-linkage, the receptor for avian influenza viruses. Experimental and epidemiologic evidence demonstrates that different subtypes of avian influenza viruses can replicate in swine (*4**–**6*). Therefore, swine can be a vessel for reassortment of human and avian influenza viruses (*7*).

The viral structure that binds to the cellular receptor is the receptor-binding site, which is located on the globular part of the hemagglutinin (HA) monomer (*8*). Based on a crystallographic model, the receptor-binding site of the H3 subtype includes conserved residues Tyr98, His193, Glu190, Trp53, and Leu194 (*8*). Two other conserved residues at positions 226 and 228 within the binding pocket determine host range specificity (*3*). Leu226 and Ser228 selectively bind to α2,6 sialosides found on human and swine cells, while Gln226 and Gly228 bind to the α2,3 sialosides found predominantly on avian cells (*3**,**9**,**10*).

Influenza viruses currently circulating in North American swine are subtypes H1N1, H3N2, and H1N2 (*11*). The classical H1N1 viruses have been circulating in the swine population since the Spanish flu pandemic of 1918 (*1*). The first SIV, A/SW/IA/15/30, was isolated in 1930 and is antigenically similar to the 1918 human influenza virus (*12*). From 1930 to 1998, classic H1N1 viruses were the predominantly isolated subtype from US swine. In 1998, a new SIV subtype H3N2 emerged and became established in the North American swine population (*13**,**14*). Genetic analysis showed that it was a triple reassortant virus containing genes from swine, human, and avian influenza viruses. The H3N2 SIV acquired the polymerase basic (PB) protein 1, HA, and neuraminidase (NA) genes from a recent human virus, the PB2 and polymerase acidic (PA) protein genes from avian viruses, and the nucleocapsid protein (NP), matrix (M), and nonstructural (NS) genes from the classic H1N1 swine virus (*13**–**16*). A year later, reassortment between the H3N2 and classic H1N1 SIV resulted in a new subtype H1N2, where the HA of the H3N2 subtype was replaced by the HA from the classic H1N1 virus (*17*). This H1N2 subtype caused respiratory disease in swine and continues to circulate in swine populations (*18*). Recently, wholly avian influenza viruses, subtypes H4N6 (*5*), H3N3, and H1N1 (*19*), from water fowl were isolated from diseased swine in Canada; however, no evidence shows that these viruses can be successfully maintained in swine populations. We identified and characterized a new SIV subtype H3N1 that may have arisen from reassortment of an H3N2 turkey isolate, a human H1N1 isolate, and currently circulating swine influenza viruses.

## Materials and Methods

### Clinical Samples

Two SIV isolates, A/SW/MI/PU243/04 (PU243) and A/SW/IN/PU542/04 (PU542), were obtained from 1 swine herd in southern Michigan and 1 in central Indiana, respectively. A/SW/MI/PU243/04 was isolated from lung tissue of a dead 7-week-old, cross-bred swine that was clinically and histologically diagnosed with viral pneumonia. A/SW/IN/PU542/04 was isolated from the nasal swab of a 14-week-old, cross-bred swine that was coughing, had dyspnea, and was lethargic. Both isolates were submitted for virus isolation to the Animal Disease Diagnostic Laboratory of Purdue University.

### Virus Isolation and Subtype Determination

Madin-Darby canine kidney (MDCK) cells were grown in Eagle's minimum essential medium supplemented with 2% fetal bovine serum. The 10% lung homogenate (PU243) and nasal swab preparation (PU542) were applied onto MDCK cells maintained in Eagle's minimum essential medium containing 4 μg/mL trypsin and 0.3% bovine serum albumin (Sigma, St. Louis, MO, USA). Cytopathic effect was observed, and the culture supernatant was tested with an HA assay using turkey erythrocytes. RNA was isolated from the supernatant of virus-infected cells by using Trizol (Invitrogen, Carlsbad, CA, USA), and the viral subtype was determined by using 2 different multiplex SIV subtype-specific reverse transcription–polymerase chain reactions (RT-PCR) (*20*). One set of 4 primers was used to differentiate H1 and H3 of HA, and another set of 4 primers was designed for N1 and N2 discrimination.

### DNA Sequencing

Two-step RT-PCR was performed by using universal primers and specific primers for influenza A viruses (*21*). The universal primers 5´-AGC AAA AGC AGG-3´ and 5´-ATG AGA AAC AAG G-3´ were used to amplify NS, M, NA, NP, and HA genes of the 2 isolates. The remaining genes, PA, PB1, and PB2, were amplified by using gene-specific primers. The primer pairs are PA F, 5´- AGC AAA AGC AGG TCA-3´; PA R, 5´-ATG AGA AAC AAG GTA CTT-3´; PB1 F, 5´-AGC AAA AGC AGG CA-3´; PB1 R, 5´-ATG AGA AAC AAG GCA TTT-3´; PB2 F, 5´-AGC AAA AGC AGG TC-3´; PB2 R, 5´-ATG AGA AAC AAG GTC GTT T-3´. RNA was reverse transcribed by using Superscript II (Invitrogen), and the cDNA was amplified by using the expand high fidelity PCR system (Roche, Indianapolis, IN, USA) according to manufacturer's instructions. The PCR products were cloned into pGEMT Easy (Promega, Madison, WI, USA). Purified plasmids containing the viral genes were sequenced by using an ABI 3100 sequencer (Applied Biosystems, Foster City, CA, USA) at the sequencing facility of the National Animal Disease Center, Agricultural Research Services, US Department of Agriculture (Ames, IA, USA). At least 4 cDNA clones of each gene were analyzed.

### Phylogenetic Analysis

Individual gene sequences were combined and edited by using Lasergene (DNASTAR, Madison, WI, USA). Megablast (National Center for Biotechnology Information, Bethesda, MD, USA) searches were performed to identify sequences with the best match to each individual gene of the 2 H3N1 viruses. Multiple alignments of DNA sequences were conducted on the complete NA gene and the HA1 region of the HA gene by using ClustalW (DNASTAR). Maximum parsimony phylogenetic trees were created by using MEGA3 (The Biodesign Institute, Tempe, AZ, USA) (*22*). The HA tree was rooted by an unrelated H4 duck influenza virus, A/duck/Alberta/28/76. An avian N2, A/chicken/CA/6643/01, represented the outgroup of the NA tree. Each tree is a consensus of 1,000 bootstrap replicates.

### Hemagglutination Inhibition (HI) Assay

HI assays were performed to determine the antigenic relationship between the 2 H3N1 viruses, the H3N2 turkey isolates (*23*), and H3N2 SIVs. The H3N2 SIVs tested in the HI assay included viruses representing 3 H3N2 clusters: cluster I, TX98 (A/SW/TX/4199-2/98); cluster II, CO99 (A/SW/CO/23619/99); and cluster III, WI99 (A/SW/WI/14094/99) and IL99 (A/SW/IL/21587/99). Swine hyperimmune sera against various H3N2 SIVs (*24*) and a ferret serum raised against an H3N2 turkey isolate were adsorbed with kaolin powder to eliminate nonspecific inhibitors. The 2 H3N1 and 4 H3N2 SIVs were tested with respective sera in a standard HI assay (*25*).

### Experimental Animal Infection

The 2 H3N1 viruses were inoculated into 10-week-old cross-bred swine in compliance with the Institutional Animal Care and Use Committee of the National Animal Disease Center. The protocol for infection is described elsewhere (*24*). Briefly, 2 groups of swine (n = 4 or 5) were infected intratracheally with 2 × 10^5^ PFU/swine of either A/SW/MI/PU243/04 or A/SW/IN/PU542/04 inoculum (total of 1 mL) prepared in embryonated eggs. Four swine were mock infected with medium only and served as controls. Five days after infection, swine were euthanized, lung lesions were scored (*24*), and bronchoalveolar lavage fluid (BALF) was collected. Sera and nasal swabs were collected the day of and 5 days after infection.

Virus load in BALF, serum samples, and nasal swabs were determined in a 96-well format (*24*). Each sample was serially diluted 10-fold and injected into a monolayer of MDCK cells. The infected cells were fixed with methanol 48 hours after infection, and an indirect immunofluorescence assay was conducted by using anti-SIV swine serum (primary antibody) and a secondary fluorescein isothiocyanate–conjugated anti-swine antiserum (Sigma). Wells were determined as either positive or negative without counting individual foci. The virus titers were determined as 50% tissue culture infective dose (TCID_50_) per milliliter.

### Detecting Swine Respiratory Pathogens

The presence of porcine reproductive and respiratory syndrome virus (PRRSV) and *Mycoplasma hyopneumoniae* in BALF was determined by using either RT-PCR or PCR assays, respectively. For PRRSV, total RNA was isolated from BALF from each swine by using the QIAamp Viral RNA mini kit (Qiagen, Valencia, CA, USA). One microgram of the extracted RNA and a primer pair specific for open reading frame 5 of PRRSV were included in a single-tube RT-PCR as described previously (*26*). To find *M. hyopneumoniae*, DNA was extracted from BALF by using the QIAamp DNA mini kit according to the manufacturer's recommendations (Qiagen). A forward primer specific for *M. hyopneumoniae* and a common reverse primer for the 16S rRNA gene were used in the PCR as previously described (*27*). A laboratory-grown *M. hyopneumoniae* DNA sample was used as a positive control. Amplified products were detected by electrophoresis on ethidium bromide–stained agarose gel.

## Results

### Virus Isolation and Subtype Determination

MDCK cells injected with a lung homogenate from swine PU243 or with the nasal swab of swine PU542 produced cytopathic effect approximately 2–3 days after infection. The supernatant agglutinated turkey erythrocytes in HA tests. Total RNA of each isolate was prepared from the supernatant of PU243- or PU542-infected MDCK cells and used as templates for the multiplex RT-PCR. Results of the multiplex RT-PCR assay specific for HA showed that both isolates were of the H3 subtype, since no H1-specific band was present. The multiplex RT-PCR specific for NA showed that both isolates were of N1 and not N2 subtype. The 2 H3N1 SIV isolates were designated A/SW/MI/PU243/04 or A/SW/IN/PU542/04. Subsequently, the RNA from the culture supernatants was used for amplification and cloning.

### Experimental Animal Infection

Four or five 10-week-old swine, negative for SIV-specific antibodies, were infected with the PU243 and PU542 H3N1 isolates. No respiratory difficulties were reported during 5 days of observation before the animals were euthanized. At necropsy, macroscopic lesions characterized by marked plum-colored, consolidated areas on lung lobes were observed. The PU243-infected swine had an average lung lesion score of ≈8%. The PU542 infected group had a milder lung lesion score of ≈3%. Control swine had no obvious lung lesions.

To determine lung replication and nasal shedding of the H3N1 viruses in swine, virus titers in the sera, nasal swabs, and BALF were evaluated. All samples from all swine in the control group as well as all samples obtained before infection were virus negative. Titers of viruses from nasal swabs and BALF 5 days after infection are shown in Table 1. Viral loads in BALF at 5 days after infection ranged from 10^6.3^ to 10^7.6^ TCID_50_/mL (mean 10^7^) and were substantially greater (p<0.05) than those in nasal swabs 5 days after infection (titer range 10^3^–10^5.8^ TCID_50_/mL, mean 10^4.7^ TCID_50_/mL). Sera from infected swine collected 5 days after infection were virus negative.

**Table 1:** Virus titers in nasal swabs and BALF from experimentally infected pigs 5 days after infection*

Inoculum/pig no.	A/SW/MI/PU243/04 (TCID_50_/mL)					A/SW/MI/PU542/04 (TCID_50_/mL)			
	108	109	111	114	127	3	110	112	113
Nasal swab	10^4.3^	10^4.7^	10^5^	10^3^	10^5.8^	10^5.8^	10^5.6^	10^4.5^	10^4.1^
BALF	10^6.6^	>10^7.3^	10^7^	>10^7.6^	10^6.3^	>10^7.5^	10^6.5^	10^7.6^	10^6.9^

*BALF, bronchoalveolar lavage fluid; 50%TCID_50_, 50% tissue culture infective dose.

Other respiratory pathogens of swine that might produce lung lesions similar to SIV were not found in BALF of infected swine. The result of a PCR specific for the 16S RNA of *M. hyopneumoniae* showed that BALF from all swine were negative. Similarly, BALF did not contain PRRSV nucleic acids. These results eliminated the possibility that swine might have been infected with *M. hyopneumoniae* or PRRSV.

### Sequence Analysis

Comparison of individual gene sequences of the 2 H3N1 SIVs showed that the identities ranged from 92.3% to 99.3% at the nucleotide level. The M gene is the most conserved, while the HA and NA genes are more variable with identities of 96.5% and 92.3% at the nucleotide level and 95.6% and 92.3% at the amino acid level, respectively. The similarity among the remaining 5 genes of the 2 isolates is >98%. Table 2 shows results obtained from Megablast analyses, which searched for sequences in the GenBank with the best match to each individual gene of both H3N1 SIVs. The HA of both H3N1s has the highest similarity with the HA of an H3N2 virus isolated from a turkey (A/TK/NC/12344/03). The NA sequence of both H3N1s is closely related to the NA of a human H1N1 isolate (A/WI/10/98) (*28*). The remaining 5 genes of both isolates are closely related to respective genes found in currently circulating H3N2 and H1N2 SIVs (Table 2). The M gene of PU243 isolate is most similar to a turkey isolate (A/TK/NC/12344/03) while the M gene of PU542 is similar to an H1N2 SIV (A/SW/IN/14810-S/01).

**Table 2:** Results of Megablast nucleotide analyses of influenza A viruses with the best match of each gene with the H3N1 swine influenza viruses*

Gene	A/SW/MI/PU243/04			A/SW/IN/PU542/04		
	Virus	Subtype	% identity	Virus	Subtype	% identity
PB2	A/SW/IL/10084/01	H1N2	98.20	A/SW/IL/10084/01	H1N2	98.20
PB1	A/SW/IA/930/01	H1N2	98.37	A/SW/IA/930/01	H1N2	97.93
PA	A/SW/IA/569/99	H3N2	97.21	A/SW/IA/569/99	H3N2	97.02
HA	A/TK/NC/12344/03	H3N2	96.91	A/TK/NC/12344/03	H3N2	97.42
NP	A/SW/OH/891/01	H1N2	98.96	A/SW/OH/891/01	H1N2	98.46
NA	A/WI/10/98	H1N1	95.54	A/WI/10/98	H1N1	93.78
M	A/TK/NC/12344/03	H3N2	98.78	A/SW/IN/14810-S/01	H1N2	99.48
NS	A/SW/IN/14810-S/01	H1N2	99.24	A/SW/IN/14810-S/01	H1N2	99.05

*Accession numbers: A/SW/MI/PU243/04 PB2 (DQ150422), PB1 (DQ150423), PA (DQ150424), HA (DQ150425), NP (DQ150426), NA (DQ150427), M (DQ150428), and NS (DQ150429); A/SW/IN/PU542/04 PB2 (DQ150430), PB1 (DQ150431), PA (DQ150432), HA (DQ150433), NP (DQ150434), NA (DQ150435), M (DQ150436), and NS (DQ150437); A/SW/IL/10084/01 PB2 (AF455738); A/SW/IA/930/01 PB1(AF455727); A/SW/IA/569/99 PA (AF251425); A/TK/NC/12344/03 HA (AY779253) and M (AY779257); A/SW/OH/891/01 NP (AF455699); A/WI/10/98 NA (AF342820); A/SW/IN/14810-S/01 M (AY060071) and NS (AY060136).

### Phylogenetic Analyses

**Figure 1:**
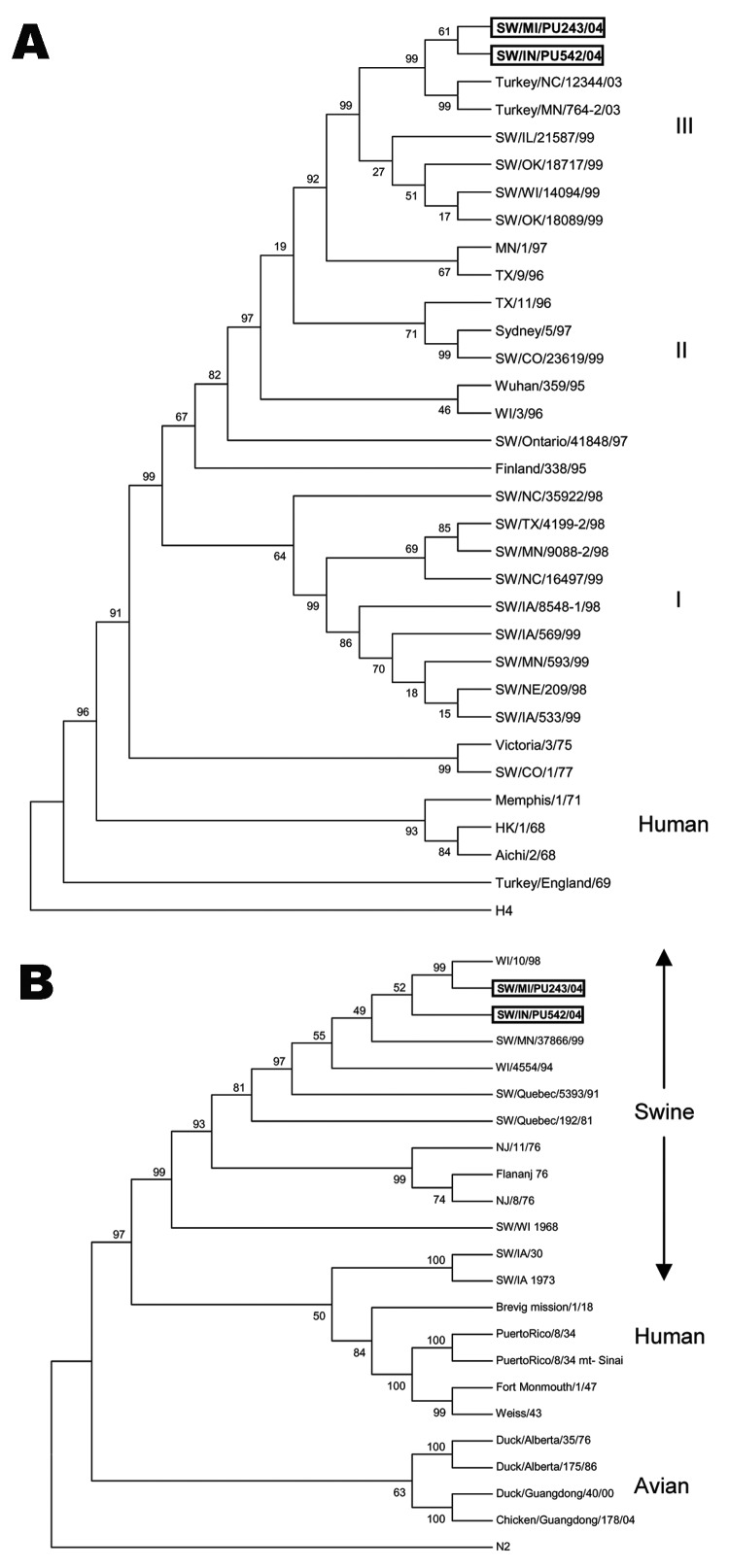
Genetic relationships of the hemagglutinin (HA) 1 region of HA gene and neuraminidase (NA) gene of the H3N1 swine influenza viruses (SIVs) with other influenza viruses. The tree was created by maximum parsimony method and bootstrapped with 1,000 replicates. The bootstrap numbers are given for each node. A) Phylogenetic trees demonstrating genetic relationship of the closely related H3N2 turkey isolates, recent H3N2 SIVs, and human H3N2s. The tree was created from the HA1 region of HA and rooted to an unrelated sequence, the H4 HA of A/duck/Alberta/28/76. B) Phylogenetic analysis of the N1 subtype of NA genes of human, swine, and avian influenza viruses. N2 of A/Chicken/CA/6643/01 was used as an outgroup sequence.

Maximum parsimony analysis of the HA1 region of the H3 subtype of recent North American SIVs separates these subtypes into 3 clusters as previously reported (Figure 1A) (*16*). Both H3N1 SIVs are closely related to 2 H3N2 turkey isolates, A/TK/NC/16108/03 and A/TK/MN/764/03, within cluster III. Branch length between the H3N1 viruses and the turkey H3N2 viruses is shorter than between the H3N1 SIVs and the swine H3N2 isolates.

Phylogenetic analysis of the N1 subtype of NA separates the sequences into 3 groups: swine, human, and avian. Both human and swine N1s share a common ancestor; however, they are placed in different clusters (Figure 1B). The N1s of the 1930 and 1973 SIVs were placed near the root of the human cluster. The human influenza isolates within the swine group were obtained from humans infected with swine viruses. The NAs of both H3N1 SIVs are placed into the swine cluster and are most closely related to an H1N1 human virus, A/WI/10/98. The A/SW/IN/PU243/04 and the 1998 H1N1 human isolate were placed in similar root at a 99% level.

### Antigenic Relationship of Swine and Turkey H3 Subtype Viruses

Cross-reactivity between the H3N1 SIVs, H3N2 turkey isolates, and H3N2 SIVs representing 3 genetic clusters (*16*) were tested in HI assays. The results showed that neither H3N1 isolate reacted with antibodies raised against H3N2 swine viruses representing cluster II and III at a 1:10 dilution, the lowest dilution tested. They reacted poorly with a serum raised against the cluster I TX98 SIV, with an HI titer of 20. PU243 reacted with a ferret serum raised against the H3N2 turkey isolate with a low HI titer of 40. PU542 reacted weakly with the same ferret serum with an HI titer of 20. Antisera from swine infected with each H3N1 virus showed weak reactivity to a H3N2 cluster I SIV (TX98) with an HI titer of 10 and cluster III SIVs (WI99 and IL99) with HI titers of 20 and moderate reactivity to a cluster II SIV (CO99) with HI titers of 80 for PU243 and 40 for PU542.

### Receptor-binding Site

**Figure 2:**
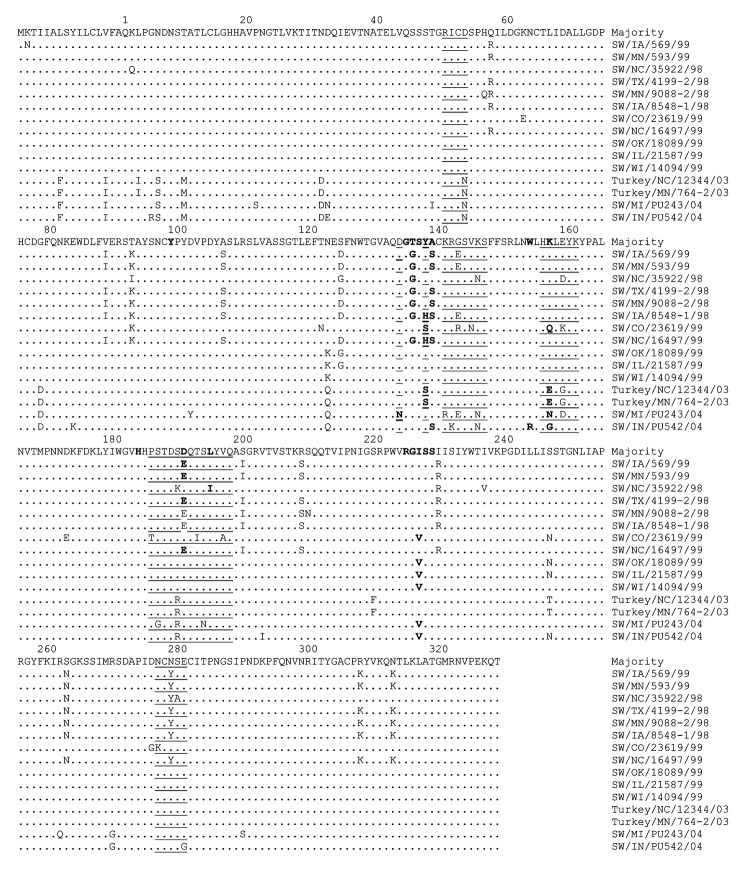
Alignment of deduced amino acid sequences within the hemagglutinin (HA) 1 region of HA genes of H3N2 swine influenza viruses (SIVs), H3N2 turkey isolates, and H3N1 SIVs. The amino acid sequence represents the consensus sequence, and the amino acid at position 1 is the first amino acid following the signal peptide (*37*). Dots represent amino acids similar to the consensus. Note that according to H3 structure (*37*), the residues representing the antigenic sites are underlined and the receptor binding sites are in **boldface**. The alignment shows that PU243 and PU542 may have emerged from the H3N2 turkey isolates. The residues within the receptor-binding site are relatively conserved.

Critical amino acid positions within the receptor-binding site of the H3 subtype of swine and turkey viruses are shown in Figure 2. Most of the residues are highly conserved, especially those associated with the sialoside receptor-binding region, Tyr98, Trp153, His183, Glu190, and Leu194 (*29**,**30*), and the residues (amino acids 226 and 228) responsible for host range specificity (*3**,**10*). All isolates have Tyr98, Trp153 (with the exception of H3N1 PU542), His183, Asp190 or Glu190, Leu194 (with the exception of H3N2 TX98), and Ser228. Residue 226 of the H3 subtype of SIVs and the H3N2 turkey viruses is either Ile or Val instead of Leu (Figure 2).

## Discussion

Although influenza viruses show host-range–specific characteristics, interspecies transmission of influenza viruses has been well documented (*1*). Infection of turkeys with swine influenza viruses seems to be common, and influenza viruses isolated from turkeys indicated that 73% of turkey influenza viruses contained genes of swine origin (*31*). Influenza viruses antigenically similar to the classic H1N1 swine virus were found to infect and produce diseases in different turkey herds (*32**–**34*). Recently, an influenza virus containing 8 genes closely related to those of A/SW/IN/9K035/99 H1N2 caused an outbreak in a turkey flock from Missouri (*35*). Thus far, transmission of turkey viruses to swine populations has not been reported.

SIV subtype H3N1 viruses were previously isolated in Taiwanese swine; these viruses most likely acquired the HA from a human H3N2 isolate and the NA from an H1N1 SIV circulating in Taiwan (*36*). The novel H3N1 SIVs reported here contain HA genes highly similar to those of recently reported H3N2 turkey isolates. These H3N2 turkey isolates, A/TK/NC/16108/03 and A/TK/MN/764/03, were most likely swine viruses, which infected and caused disease in turkeys (*23*). Phylogenetic analysis of the HA1 region of the HA gene placed the H3N1 SIVs at a similar root to the turkey isolates. Additionally, branch lengths of the H3N1 SIVs and the turkey isolates are shorter than those between the H3N1 viruses and the swine H3N2 viruses. This finding suggests that the H3N1 SIVs may have acquired their HA from a virus similar to the H3N2 turkey isolate; this finding could indicate interspecies transmission from turkeys to swine. Subsequently, the swine H3N1s have diverged separately from the turkey H3N2s.

Maximum parsimony analysis of the NA gene separates human, avian, and swine clusters as previously reported (*37*). The NA of the H3N1 SIVs is placed into the swine cluster. A/SW/MI/PU243/04 shares a similar root with the human H1N1 isolate, WI/10/98, at the 99% level. This finding strongly suggests that both viruses have a common ancestor; however, the H3N1 swine virus may have evolved from the WI/10/98 H1N1 or similar human isolates. Although the 2 H3N1 SIVs were isolated from 2 separate herds, they may have evolved from a similar ancestor. Both HA and NA phylogenetic analyses placed the 2 isolates into different branches at 61% and 52% bootstraps, respectively. Both may have originated from a similar reassortant event and continued diverging from each other. Analysis of the deduced amino acid sequence (Figure 2) also supports this assumption.

Residues mainly responsible for sialyl α2,6-galactose specificity are Leu226 and Ser228 (*3**,**10*). Leu226 is not in contact with the sialoside but changes in this position alter the conformation of the binding pocket (*30*). The space-filling model of the H3 HA complex with a receptor analog showed that Leu226 is in close proximity to the Van der Waal space of C6 of the galactose (*29*). The H3N1 and H3N2 SIV sequences, including the H3N2 turkey viruses, contain all Ser228 but Ile226 or Val226 instead of Leu226 in their HA1 molecules. H3 subtypes of human influenza viruses isolated from Japan and China during 1994 and 1995 also contain Ile226 instead of Leu226 (*38*). Leu, Ile, and Val are similar neutral nonpolar amino acids; substitution between them most likely maintains hydrophobic interactions and proper conformation of the binding pocket. In contrast, Gln226 and Gly228 are normally found in the HA1 molecule of avian viruses (*10*). Gln is classified as a hydrophilic amino acid, and its amino acid structure is different from Leu, Ile, or Val. Ser and Gly are classified into different groups of amino acids; they possess different charges and structure. The H3 turkey HA still maintains Ile226 and Ser228 similar to that of swine viruses, indicating that they maintained their ability to infect swine and possibly humans, despite replicating in an avian host. Whether influenza virus receptors in turkeys are different from those in other avian species is not known.

Why it took ≈6 years for H3N1 SIVs to emerge in US swine where H3N2 and H1N1 viruses have been cocirculating since 1998 is not known. Reassortant H1N2 and H1N1 SIVs were isolated shortly after the 1998 introduction of the H3N2 viruses into US swine. A certain constellation of the HA and NA surface molecules was necessary to create a successful H3N1 reassortant, since optimal balance between NA activity and HA affinity to the sialoside receptor is crucial for effective influenza virus infections (*39**–**41*).

Our study showed that the H3N1 SIVs can replicate in the respiratory tract of swine and are shed in nasal secretions. In this study, investigations on virus transmissibility in which contact animals are housed together with infected animals were not performed. Therefore, whether these 2 H3N1 SIVs will be transmitted efficiently in the field situation requires further experimental and epidemiologic studies. However, our results underline the scenario in which swine can be a mixing vessel for human, swine, and avian influenza viruses to create new reassortants that may be dangerous to human health. Turkeys are more susceptible to influenza viruses from waterfowl than are other domestic poultry (*42*), and a high degree of genetic reassortment most likely occurs in domestic turkeys (*31*). This finding may indicate that influenza A viruses could sequentially acquire new genes during transmission from waterfowl via turkey to swine and humans.
